# Receptor-Mediated Bioassay Reflects Dynamic Change of Glucose-Dependent Insulinotropic Polypeptide by Dipeptidyl Peptidase 4 Inhibitor Treatment in Subjects With Type 2 Diabetes

**DOI:** 10.3389/fendo.2020.00214

**Published:** 2020-04-24

**Authors:** Tsuyoshi Yanagimachi, Yukihiro Fujita, Yasutaka Takeda, Jun Honjo, Hiroki Yokoyama, Masakazu Haneda

**Affiliations:** ^1^Division of Metabolism and Biosystemic Science, Department of Internal Medicine, Asahikawa Medical University, Asahikawa, Japan; ^2^Department of Diabetology, Endocrinology and Nephrology, Department of Internal Medicine, Shiga University of Medical Science, Otsu, Japan; ^3^Jiyugaoka Medical Clinic, Internal Medicine, Obihiro, Japan

**Keywords:** glucose-dependent insulinotropic polypeptide, glucagon-like peptide 1, glucagon, receptor-mediated bioassay, dipeptidyl peptidase 4

## Abstract

**Objective:** We recently observed a greater increase in plasma levels of bioactive glucose-dependent insulinotropic polypeptide (GIP) than glucagon-like peptide 1 (GLP-1) using the receptor-mediated bioassays in the subjects with normal glycemic tolerance (NGT) treated with dipeptidyl peptidase 4 (DPP-4) inhibitors, which may be unappreciated using conventional enzyme-linked immunosorbent assays (ELISAs) during oral glucose tolerance test. Thus, we determined incretin levels in addition to glucagon level using the bioassays in type 2 diabetes mellitus (T2DM) subjects with or without treatment of DPP-4 inhibitor, to evaluate whether these assays can accurately measure bioactivity of these peptides.

**Methods:** We performed single meal tolerance test (MTT) by using a cookie meal (carbohydrate 75.0 g, protein 8.0 g, fat 28.5 g) in the subjects with NGT (*n* = 9), the subjects with T2DM treated without DPP-4 inhibitor (*n* = 7) and the subjects with T2DM treated with DPP-4 inhibitor (*n* = 10). All subjects fasted for 10–12 h before the MTT, and blood samples were collected at 0, 30, 60, and 120 min. We used the cell lines stably cotransfected with human-form GIP, GLP-1 or glucagon receptor, and a cyclic adenosine monophosphate–inducible luciferase expression construct for the bioassays. We measured active GIP, active GLP-1, and glucagon by the bioassays. To evaluate the efficacy of bioassay, we measured identical samples via ELISA kits.

**Results:** During the single MTT study, postprandial active GIP _*bioassay*_ levels of T2DM with DPP-4 inhibitor treatment were drastically higher than those of NGT and T2DM without DPP-4 inhibitor, although the DPP-4 inhibitor-treated group showed moderate increase of active GIP_ELISA_ and active GLP-1_*bioassay*_, while active GLP-1_*bioassay*_ levels of T2DM subjects without DPP-4 inhibitor were comparable to those of NGT subjects. During the serial MTT, administration of DPP-4 inhibitor significantly increased active GIP_*bioassay*_ levels, but not active GLP-1_*bioassay*_.

**Conclusions:** In comparison to conventional ELISA, receptor-mediated bioassay reflects dynamic change of GIP polypeptide by DPP-4 inhibitor treatment in subjects with type 2 diabetes.

## Introduction

Glucose-dependent insulinotropic polypeptide (GIP) and glucagon-like peptide 1 (GLP-1) are incretin hormones, released from the intestine by oral ingestion of various nutrients (1, 2). They stimulate insulin secretion from beta cells in the islet (1, 2) and are cleaved rapidly after secretion by dipeptidyl peptidase 4 (DPP-4) (3). Pro-GIP and proglucagon are processed to GIP and GLP-1 in the gut by prohormone convertase (PC) 1/3, respectively (4–6). Glucose-dependent insulinotropic polypeptide consists of 42 amino acids and is secreted from K-cells in the upper small intestine (1, 2, 6).

Interestingly, it is reported that pro-GIP is processed to a short-form GIP (1–30)_NH2_ in the pancreatic alpha cells and the gastrointestinal tract by PC2 (5, 7), and this form has insulinotropic activity almost equivalent to GIP(1–42) (7). Further, proglucagon is alternatively modified to GLP-1 in the pancreatic alpha cells during some metabolic states by PC1/3 (8).

Recently, several enzyme-linked immunosorbent assay (ELISA) kits have been developed to measure specifically active GIP and GLP-1, but it is unclear if these kits can accurately quantify all bioactive forms. Indeed, short-form GIP(1–30)_NH2_ could never be detected by total and active GIP ELISA kits; however, we confirmed that it increased receptor-mediated cyclic adenosine monophosphate (cAMP) production in a concentration-dependent manner in our cell-based receptor-mediated bioassay (9). Additionally, we reported that plasma active GIP bioassay levels were boosted by DPP-4 inhibitor administrated in non-diabetic subjects during oral glucose tolerance test (OGTT), whereas total GIP_ELISA_ and active GIP_ELISA_ levels indicated only mild increases (9). Therefore, commercial ELISA kits may underestimate incretin activities particularly GIP under DPP-4 inhibitor treatment.

In the current study, we here determined active GIP, GLP-1, and glucagon levels in type 2 diabetes mellitus (T2DM) subjects with or without DPP-4 inhibitor treatment to characterize the cell-based, receptor-mediated bioassays in comparison to immunoassays.

## Materials and Methods

### Subjects and Study Protocol

We recruited Japanese subjects with normal glucose tolerance (NGT: *n* = 9) and T2DM subjects [without DPP-4 inhibitor treatment: *n* = 7, with DPP-4 inhibitor treatment: *n* = 10 (sitagliptin: *n* = 6, vildagliptin: *n* = 2, anagliptin: *n* = 1, alogliptin: *n* = 1)]. The written informed consent was obtained from all participants. The study was performed in accordance with the Declaration of Helsinki, and the research protocol was approved by the Research Ethics Committee of Asahikawa Medical University (approved protocol no. 1074-2). The clinical characteristics of the subjects are shown in Table 1.

**Table 1:** Participants characteristics.

	**NGT**	**T2DM**	
		**DPP-4 inhibitor (–)**	**DPP-4 inhibitor (+)**
N (male/female)	9 (3/6)	7 (6/1)	10 (6/4)
Glucose-lowering agents (excluding DPP-4 inhibitors)	—	5	9
Insulin	—	4	2
Sulfonylurea	—	—	1
Metformin	—	2	8
Pioglitazone	—	1	1
Age (year)	65.4 ± 4.5	66.6 ± 3.6	65.8 ± 3.2
BMI (kg/m^2^)	26.1 ± 1.5	25.0 ± 1.0	27.0 ± 1.3
eGFR (mL/min per 1.73 m^2^)	69.5 ± 3.3	80.9 ± 9.2	63.9 ± 5.4
HbA_1c_ (%)	5.7 ± 0.1	8.6 ± 0.9	6.7 ± 0.2

*Date are presented as means ± SEM. Age, BMI, eGFR: 1-way ANOVA by the Tukey post hoc test. HbA_1c_, Mann–Whitney U test [DPP-4 inhibitor (–) vs. DPP-4 inhibitor (+)].BMI, body mass index; DPP-4, dipeptidyl peptidase-4; eGFR, estimated glomerular filtration rate; HbA_1c_, hemoglobin A_1c_; NGT, normal glycemic control; T2DM, type 2 diabetes*.

We performed single meal tolerance test (MTT) by using a cookie meal (Meal test C^TM^; Saraya Corporation, Osaka, Japan; 592 kcal, carbohydrate 75.0 g, protein 8.0 g, fat 28.5 g) from 2014 to 2017 (10–12). All subjects were fasted for 10–12 h before the MTT. We collected blood samples at 0, 30, 60, and 120 min using blood collection tubes containing DPP-4 inhibitor (P800; BD, Tokyo, Japan).

We excluded subjects with type 1 diabetes, malignancy, renal failure, pregnancy, other endocrine disorders, and steroid medication.

The plasma samples were separated by centrifugation (3,000 revolutions/min, 15 min, 4°C) and then stored at −80°C until assays.

### Assays

Plasma GIP, GLP-1, and glucagon levels were measured by ELISA and our bioassays employing HEK293 cell lines stably cotransfected with human forms of the GIP receptor, GLP-1 receptor, or glucagon receptor (GCGR), respectively, and a cAMP-inducible luciferase (CRE-Luc) expression construct (9). For ELISAs, we used total GIP (EZHGIP-54K; Millipore, Tokyo, Japan), active GIP (#27201; IBL, Fujioka, Japan), total GLP-1 (EZGLP1T-36K; Millipore), and glucagon (#10-1281-01; Mercodia, Uppsala, Sweden). Each assay was performed with previously unthawed plasma samples.

For the bioassays, we utilized HEK293 cell lines stably cotransfected with human-form GIP, GLP-1, or glucagon receptor and a CRE-Luc expression construct. The cell lines were cultured in DMEM (25 mmol/L glucose) with 10% (vol/vol) fetal bovine serum (FBS) (MP Biomedicals, Santa Ana, CA, USA), 100 IU/mL penicillin, and 100 μg/mL streptomycin (Invitrogen, Tokyo, Japan) at 37°C in 5% CO_2_. Cells were harvested overnight on 96-well plates (100,000 cells/well). Media was then replaced with samples or various concentrations of synthetic peptides [GIP bioassay: GIP(1–42), GIP(1–30)_NH2_, GLP-1(7–36)_NH2_, glucagon(1–29) and oxyntomodulin; 10^−13^–10^−7^ mol/L, GLP-1 bioassay: GLP-1(7–36)_NH2_, GIP(1–42), GIP(1–30)_NH2_, glucagon(1–29), and oxyntomodulin; 10^−13^–10^−9^ mol/L, glucagon bioassay: glucagon(1–29), oxyntomodulin, GIP(1–42), GIP(1–30)_NH2_ and GLP-1(7–36)_NH2_; 10^−13^–10^−9^ mol/L] that were diluted in Krebs–Ringer buffer (KRB) (pH 7.4) containing 0.5% (wt/vol) bovine serum albumin (Sigma-Aldrich, Tokyo, Japan) and incubated for 5 h at 37°C in 5% CO_2_. We diluted plasma samples with KRB before active GIP, active GLP-1, and glucagon measurement by bioassay (GIP: 20-fold dilution, GLP-1: 50-fold dilution, glucagon: 20-fold dilution). After incubation, we measured luciferase activity with the Bright-Glo Assay Kit (Promega, Madison, WI, USA) using Thermo Scientific Appliskan (Thermo Fisher Scientific, Waltham, MA, USA) according to the manufacturer's instructions and calculated the hormone concentrations as previously described (9).

### Peptides and Enzyme

GIP(1–42), GLP-1(7–36)_NH2_, and glucagon(1–29) were purchased from Peptide Institute (Osaka, Japan). Glucose-dependent insulinotropic polypeptide(1–30)_NH2_ was purchased from Phoenix Pharmaceuticals (Burlingame, CA, USA). Oxyntomodulin was purchased from American Peptide (Sunnyvale, CA, USA). Dipeptidyl peptidase 4 inhibitor was purchased from Millipore.

### Statistical Analysis

Data are expressed as mean ± SEM. Statistical analysis was performed by Mann–Whitney *U* test, one-way analysis of variance (ANOVA) (followed by the Tukey *post hoc* test or two-way repeated ANOVA followed by the Bonferroni *post hoc* test. Data were analyzed using the commercial software (Prism 5; GraphPad, San Diego, CA, USA), and *p* < 0.05 was considered significant.

## Results

### The Receptor-Mediated Bioassay and ELISA

**Figure 1:**
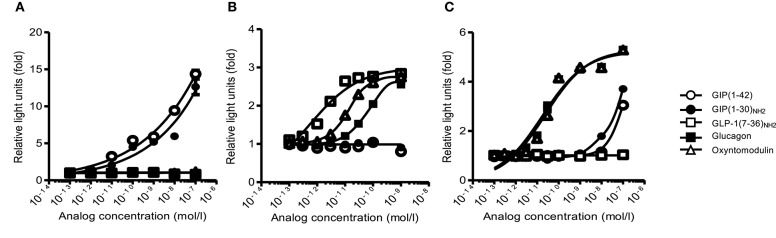
The receptor-mediated bioassay. The responsiveness and specificity of **(A)** GIP, **(B)** GLP-1, and **(C)** glucagon receptor-mediated bioassays with GIP, GLP-1, glucagon, and oxyntomodulin peptides. White circle, GIP(1–42); black circle, GIP(1–30)_NH2_; white square, GLP−1(7-36)_NH2_; black square, glucagon; white triangle, oxyntomodulin. Data are presented as means ± SEM. GIP, glucose-dependent insulinotropic polypeptide; GLP-1, glucagon-like peptide 1.

To evaluate the specificity and the characteristics of the bioassays, we examined their responsiveness with several synthetic glucagon-related peptides. In the GIP bioassay, GIP(1–42) and GIP(1–30)_NH2_ almost equivalently increased luciferase activity in a concentration-dependent manner. Glucagon-like peptide 1(7–36)_NH2_, glucagon, and oxyntomodulin did not increase luciferase activity at any concentration (Figure 1A). In the GLP-1 bioassay, GLP-1(7–36)_NH2_ increased luciferase activity in a concentration-dependent manner. Glucagon and oxyntomodulin induced luciferase activity at concentrations >~10^−11^ and 3 × 10^−12^ mol/L, respectively (Figure 1B). In the glucagon bioassay, glucagon and oxyntomodulin increased luciferase activity in a concentration-dependent manner, and they showed almost the equivalent bioactivities. Glucose-dependent insulinotropic polypeptide induced luciferase activity at concentrations >~10^−8^ mol/L (Figure 1C).

Neither total nor active GIP ELISA kits detected GIP(1–30)_NH2_ as we previously reported (9). Glucagon ELISA kit did not detect GIP(1–42) and GIP(1–30)_NH2_. The cross-reactivity against oxyntomodulin (measurement range, 1–500 pmol/L) was ~5% (Supplementary Figure 1).

### High Active GIP Levels by Bioassay in T2DM Subjects Under DPP-4 Inhibitor Treatment

**Figure 2:**
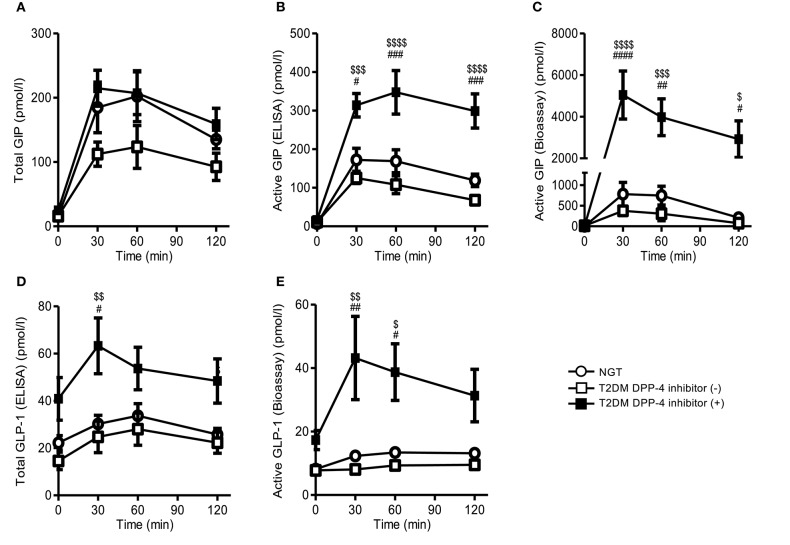
Plasma GIP and GLP-1 levels of NGT and T2DM subjects during the MTT. We performed single MTT by using a cookie meal and measured plasma total GIP **(A)**, active GIP (ELISA) **(B)**, active GIP (bioassay) **(C)**, total GLP-1 **(D)**, and active GLP-1 (bioassay) **(E)**. White circle, NGT; white square, T2DM without DPP-4 inhibitor; black square, T2DM with DPP-4 inhibitor. Data are presented as means ± SEM. ^#$^*p* < 0.05, ^##$$^*p* < 0.01, ^###$$$^*p* < 0.001, ^####$$$$^*p* < 0.0001. ^#^indicates NGT vs. DPP-4 inhibitor (+), ^$^is DPP-4 inhibitor (–) vs. DPP-4 inhibitor (+).DPP-4, dipeptidyl peptidase-4; GIP, glucose-dependent insulinotropic polypeptide; GLP-1, glucagon-like peptide 1; NGT, normal glycemic control; T2DM, type 2 diabetes.

During MTT study, fasting and postprandial total GIP ELISA levels in T2DM subjects with DPP-4 inhibitor treatment were comparable to NGT, but the postprandial levels of T2DM subjects without DPP-4 inhibitor tended to be lower than those of T2DM subjects with DPP-4 inhibitor, but not significantly (Figure 2A). Postprandial active GIP_*bioassay*_ and active GIP_ELISA_ levels in T2DM subjects with DPP-4 inhibitor were significantly higher than those in T2DM without DPP-4 inhibitor (Figures 2B,C). In contrast, postprandial active GIP_*bioassay*_ level in T2DM subjects without DPP-4 inhibitor tended to be lower than those in NGT, as well as total GIP _ELISA_ and active GIP _ELISA_ levels (Figures 2A–C). Postprandial active GLP-1_*bioassay*_ and total GLP-1_ELISA_ levels in T2DM with DPP-4 inhibitor were significantly higher than those of T2DM without DPP-4 inhibitor and NGT (Figures 2D,E). Fasting and postprandial active GLP-1_*bioassay*_ levels of T2DM subjects without DPP-4 inhibitor were comparable to those of NGT subjects (Figures 2D,E).

Among the T2DM subjects with DPP-4 inhibitor treatment, active GIP_*bioassay*_ levels were increased ~15-fold at 30 min compared to without DPP-4 inhibitor (Figure 2C). In contrast, total GIP_ELISA_ and active GIP_ELISA_ at 30 min were only 2- and 2.5-fold greater than that of T2DM without DPP-4 inhibitor, respectively (Figures 2A,B). Moreover, active GLP-1_*bioassay*_ did not show such large changes as in GIP via the administration of DPP-4 inhibitor. Total GLP-1_ELISA_ and active GLP-1_*bioassay*_ levels at 30 min were only 2.6- and 5.3-fold greater than that of T2DM without DPP-4 inhibitor treatment, respectively (Figures 2D,E).

Glucagon levels tended to increase after MTT in T2DM subjects. Glucagon _*bioassay*_, but not glucagon _ELISA_, levels in T2DM showed significantly higher than those of NGT (Supplementary Figures 2A,B).

## Discussion

In clinical settings, plasma GIP and GLP-1 are conventionally measured by ELISA. These ELISA kits have been developed to measure specifically total/active GIP or GLP-1. However, these kits might be expected with difficulty to distinguish similar amino-acid sequence or multiple isoforms. We previously showed that some GIP commercial ELISA kits could not detect short-form GIP(1–30)_NH2_ (9). Therefore, all active forms may not be evaluated by immunological assays. Additionally, we confirmed that oxyntomodulin can stimulate GLP-1 based on our receptor-mediated bioassay. In physiological aspects, we propose that receptor-mediated bioassays are beneficial to reflect actions of each peptide and can explain the gaps between hormone action and plasma levels measured by ELISA. It is apparently important to measure concentration of peptides such as glucagon and glucagon-related peptides, specifically, and respectively. However, it is also crucial in physiological aspects to measure accurate bioactivity of glucagon-receptor activation, because it is reported that oxyntomodulin also stimulates glucagon receptor, as well as GLP-1 receptor. Indeed, our bioassay can detect glucagon-receptor activation by oxyntomodulin. Therefore, our bioassay is more useful than conventional ELISA in respect of assessing bioactivity.

In our current MMT study, T2DM subjects with DPP-4 inhibitor treatment presented significantly elevated postprandial active GIP_*bioassay*_ than those of T2DM without the treatment, similarly to our previous study examined in NGT subjects (9). Active GIP_ELISA_ and total GIP_ELISA_ levels also were increased, but those increments were smaller than that in active GIP_*bioassay*_ under the DPP-4 inhibitor treatment. Glucose-dependent insulinotropic polypeptide(1–42) is secreted from the gut K cells and processed from pro-GIP by PC1/3 (4, 5). Additionally, pro-GIP is processed to, another GIP isoform, short-form GIP(1–30)_NH2_ in the pancreatic alpha cells and in the gut by PC2 (7). On the other hand, GIP(1–42) and GIP(1–30)_NH2_ are almost equivalently cleaved by DPP-4 in a few minutes after secretions. We presume that the receptor-mediated bioassay can reflect to dynamic change of active GIP by DPP-4 inhibitor, because the bioassay, but not ELISA, was able to detect both GIP(1–42) and GIP(1–30)_NH2_. Therefore, it can be great advantage to measure GIP by our bioassay, instead of conventional ELISA kits.

Glucagon-like peptide 1 also has two active forms, GLP-1(7–36)_NH2_ and GLP-1(7–37). Here, active GLP-1 _*bioassay*_ was slightly enhanced by DPP-4 inhibitor, which was comparably assessed by the bioassay or ELISA. Collectively, DPP-4 inhibitor might contribute to enhancing bioactive GIP more extensively than GLP-1.

The present study demonstrated that GIP and GLP-1 in T2DM without DPP-4 inhibitor were lower than those of NGT, although previous reports indicated that plasma GIP and GLP-1 levels in subjects with T2DM are increased, comparable, or decreased compared to those in NGT subjects based on ELISA assays (13, 14). The previous reports were variable because those studies were performed occasionally with OGTT or MTT. In fact, the differences of nutrients can differently affect GIP and GLP-1 secretions. Glucose-dependent insulinotropic polypeptide and GLP-1 secretions are stimulated by glucose (15, 16). Additionally, other nutrients such as fat and amino acids also promote incretin secretions (16). Especially GIP secretion is strongly enhanced by fat (17, 18). In the current study, we performed MTT by using the cookie meal. Unlike OGTT, the cookie meal is composed of carbohydrate, protein, and fat. Other MTT study also used test meal composed of various nutrients (19, 20). Therefore, we presume that GIP/GLP-1 secretion is inconsistent during OGTT or MTT.

There are several limitations in this study. First, our results were based on a small number of subjects. Second, characteristics of the subjects were relatively diverse. Therefore, big cohort studies with sufficient number of subjects are necessary to be examined for further understandings. Additionally, our bioassays can detect all agonist to the receptors, and we estimated bioactivities only through PKA-cAMP activities signaling pathways. However, other pathways besides PKA-cAMP pathway might be involved in signaling after receptor biding, contributing physiological actions. Thus, it is still unclear whether luciferase activities in receptor-mediated bioassay represent all actions *in vivo*. Finally, Miyachi et al. presented the quantitative method for GIP using Liquid Chromatography-Triple Mass Spectrometry (LC-MS/MS/MS) (21). The technique may be able to detect both GIP(1–42) and GIP(1–30)_NH2_. LC-MS/MS/MS could provide us more outcomes in addition to our results. In addition, we are now developing new GIP ELISA system specifically to assess GIP(1–30)_NH2_ concentration. We expect further consequences can be obtained through our new system.

In conclusion, total and active GIP ELISA kits did not reflect all bioactive GIP increased by DPP-4 inhibitor treatment. In comparison to conventional ELISA, receptor-mediated bioassay reflects dynamic change of GIP by DPP-4 inhibitor treatment in subjects with type 2 diabetes.

## Data Availability Statement

The datasets generated for this study are available on request to the corresponding author.

## Ethics Statement

The studies involving human participants were reviewed and approved by The Research Ethics Committee of Asahikawa Medical University. The patients/participants provided their written informed consent to participate in this study.

## Author Contributions

TY and YF conceived, designed the study, analyzed data, and wrote the manuscript. TY, YF, YT, JH, and HY collected data. MH contributed to the discussion, and reviewed the manuscript. All authors read and approved the final manuscript.

## Conflict of Interest

The authors declare that the research was conducted in the absence of any commercial or financial relationships that could be construed as a potential conflict of interest.

## Abbreviations

BMI: body mass index
CRE-Luc: cyclic AMP-inducible luciferase
DPP-4: dipeptidyl peptidase 4
eGFR: estimated glomerular filtration rate
GIP: glucose-dependent insulinotropic polypeptide
GLP-1: glucagon-like peptide 1
KRB: Krebs Ringer Buffer
MTT: meal tolelance test
NGT: normal glycemic control
OGTT: oral glucose tolerance test
PC: prohormone convertase
T2DM: type 2 diabetes.
