# Supplementary material for: Receptor-Mediated Bioassay Reflects Dynamic Change of Glucose-Dependent Insulinotropic Polypeptide by Dipeptidyl Peptidase 4 Inhibitor Treatment in Subjects With Type 2 Diabetes
Source: Front Endocrinol (Lausanne). 2020 Apr 24;11:214. doi: 10.3389/fendo.2020.00214 (PMC7193081; doi:10.3389/fendo.2020.00214)
Supplement: Supplementary file 1 [file Data_Sheet_1.docx]

Supplementary Material

## Supplementary Figures


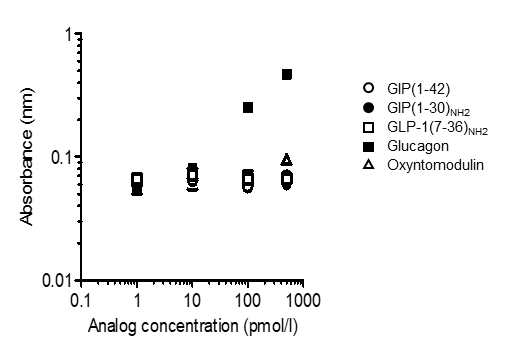


**Supplementary Figure 1.** The cross-reactivity glucagon and other glucagon related hormone using glucagon ELISA kit. Response profile for glucagon and other glucagon related peptides measured by Mercodia glucagon ELISA kit. White circle, GIP(1-42); black circle, GIP(1-30)_NH2_; white square, GLP-1(7-36)_NH2_; black square, glucagon; white triangle, oxyntomodulin. Data are presented as means ± SEM.

**Supplementary Figure 2.** Glucagon levels of NGT and T2DM subjects during the MTT. We performed single MTT by using a cookie meal and measured plasma Glucagon (ELISA) **(A)**, Glucagon (bioassay) **(B)**. White circle, NGT; white square, T2DM without DPP-4 inhibitor; black square, T2DM with DPP-4 inhibitor. Data are presented as means ± SEM. ⋆ p<0.05, ## p<0.01, ### p<0.001. ⋆ is NGT vs DPP-4 inhibitor (-), # indicates represents NGT vs DPP-4 inhibitor (+).

T2DM DPP-4 inhibitor (-)

T2DM DPP-4 inhibitor (+)

NGT

B

A
